# Current situation and influencing factors of disease uncertainty in parents of children with Sturge‒Weber syndrome: a retrospective study

**DOI:** 10.1186/s12887-023-03857-x

**Published:** 2023-02-07

**Authors:** Na Du, Yue Wu, Shanshan Xiong, Hong Ji, Lulu Huang, Wenyi Guo, Changjuan Zeng

**Affiliations:** 1 Department of Nursing, Ninth People’s Hospital, Shanghai Jiao Tong University School of Medicine, Shanghai, 200011 China; 2 Department of Ophthalmology, Ninth People’s Hospital, Shanghai Jiao Tong University School of Medicine, Shanghai, 200011 China; 3 Shanghai Key Laboratory of Orbital Diseases and Ocular Oncology, Shanghai, 200011 China; 4 Shanghai JiaoTong University School of Nursing, Shanghai, 200025 China

**Keywords:** Sturge‒Weber syndrome, Parents, Disease uncertainty, Present situation, Influence factor

## Abstract

**Background:**

Sturge Weber syndrome (SWS), can cause extensive capillary malformations on the face, head, trunk, and other parts of the body, and the eyes can also suffer optic nerve injury. Secondary glaucoma can cause blindness, which has the characteristics of a relatively hidden onset and unclear pathogenesis. The treatment of SWS secondary glaucoma has always been difficult, and due to the characteristics of the disease, there is uncertainty about the long-term efficacy and safety of various treatment methods for such patients.

**Methods:**

A total of 105 parents of children with SWS completed a self-designed general information questionnaire, a generalized anxiety questionnaire (GAD-7), a patient health questionnaire (PHQ-2), a stress perception scale (PSS-4), a simple coping scale (SCSQ) and a disease-uncertainty scale (PPUS).

**Results:**

The total uncertainty score of parents of children with SWS was 79.07 ± 13.24, and the average item score was 2.82 ± 0.47. Multiple linear regression analysis revealed that anxiety and simple coping were the main influencing factors of disease uncertainty among parents of children with SWS (*P* < 0.05).

**Conclusions:**

Parents of children with SWS exhibit a high level of disease uncertainty. Medical staff should pay attention to the source of parents' disease uncertainty and provide targeted interventions, which are of great importance in reducing parents' disease uncertainty.

## Background

Sturge‒Weber syndrome (SWS), also known as cerebral trigeminal hemangioma syndrome, is a neurocutaneous syndrome involving the facial skin, central nervous system and eyes and is often accompanied by glaucoma and facial features. Capillary malformations are distributed by the trigeminal nerve [1] with an incidence of 1/50000–1/20000 [2]. Patients with SWS secondary glaucoma may have other retinal complications [3]. In addition, focal seizures of the contralateral body may also be noted in these patients [4]. The onset of the disease is sporadic and does not have a genetic tendency. At present, it is believed that somatic mutations occur in early embryonic development [5]. SWS secondary glaucoma can cause continuous damage to the optic ganglion cells of patients and eventually lead to blindness. Similar to other types of glaucoma, SWS secondary glaucoma has the characteristics of a more hidden onset [6]. SWS secondary glaucoma patients are often in the late stage of the disease when experience eye abnormalities, and it is difficult to effectively intervene and reverse this condition. The treatment of SWS secondary glaucoma has always been difficult, and there is uncertainty about the long-term efficacy and safety of various treatment methods for such patients given the characteristics of the disease. SWS is a rare disease with multisystem involvement. The treatment cycle is long, the cost of treatment is expensive, and the treatment effect is relatively poor. As the main caregivers in the child's daily life, recovery and treatment, the parents of children with SWS are faced with many pressures. These pressures affect the parents’ quality of life and psychology and make them prone to a sense of disease uncertainty, which is associated with the child's mental health and wellbeing [7]. Disease uncertainty refers to the individual's lack of ability to judge disease-related stress events [8]. Illness uncertainty can damage family members' quality of life and physical and mental health, affect family members' role adaptation, interfere with their decision-making functions, and affect the patient's disease recovery [9, 10]. In recent years, disease uncertainty among parents has received increasing attention, but research has mainly focused on the parents of children receiving palliative care [11] or children with congenital heart disease [12] or epilepsy [13]. Parents of children with SWS have a high level of illness uncertainty, and their psychological state is not good. The illness uncertainty experienced by parents is proportional to their psychological state [14]. However, there are few reports on the disease uncertainty of parents of children with SWS because the disease is rare. However, our research center currently has the largest sample of patients with SWS reported in the world. This paper aims to initially explore the current situation of disease uncertainty in parents of children with SWS and explore its influencing factors with a view toward developing interventions for medical staff to implement. The paper aims to provide evidence for interventions that are effective in reducing parental illness uncertainty.

### Objects and methods

#### Research objects

Using convenience sampling, 105 parents of children with SWS who were treated in the Ninth People's Hospital Affiliated to Shanghai Jiaotong University School of Medicine from April 2020 to June 2021 were selected as the research subjects. The inclusion criteria were as follows: children diagnosed with Sturge–Weber syndrome 1 month or more, who had clear consciousness, and no recent history of major family accident or acute psychological trauma. The exclusion criteria were as follows: age < 18 years old; history of mental illness, depression or severe cognitive dysfunction; inability to understand the content of the test questionnaire; and refusal or lack of cooperation in completing the questionnaire.

### Research methods

#### General information questionnaire

Based on the literature research and preinterview, this questionnaire was designed by the researchers and included 14 items on topics, such as the relationship between the interviewee and the patient, age, place of residence, marital status, and level of education.

#### Generalized anxiety questionnaire (7-item Generalized Anxiety Disorder Scale,GAD-7)

The GAD-7 is a screening tool for generalized anxiety disorder and consists of seven items that ask subjects how distressed they have been according to the corresponding symptoms in the past two weeks. The responses are scored on a four-point scale ranging from 0 (not at all) to 3 (almost every day), and the total score ranges from 0 to 21. For the Chinese version of the GAD-7, a total score greater than or equal to 10 points indicates generalized anxiety [15]. The sensitivity and specificity of the GAD-7 for screening generalized anxiety disorder in general hospitals among Chinese outpatients were 86.2% and 95.5%, respectively. The Cronbach's α coefficient was 0.898, and the test–retest reliability coefficient was 0.856 [16].

#### Patient health questionnaire (PHQ-2)

The PHQ-2, which was extracted from the 9-item Patient Health Questionnaire (PHQ-9), was used to study depressive symptoms. The PHQ-2 measures the frequency of depressive mood and anhedonia over the past 2 weeks with scores ranging from 0 (not at all) to 3 (almost every day) [17]. The sensitivity and specificity of the PHQ-2 for diagnosing major depression were 86% and 78%, respectively, with a score of 2 or higher and 61% and 92% with a score 3 or higher [18]. The PHQ-2 has been verified and used in China [19].

#### Perceived stress scale (PSS-4)

The PSS-4 compiled by Cohen [20] was used to assess subjects’ stress levels and comprises four items rated on a 5-point Likert scale with scores ranging from 0 (never) to 4 (very common). Compared with the Stress Perception Scale, the PSS-4 is simpler and more intuitive. A number of confirmatory factor analysis studies on the PSS-4 scale have found that it contains two factor structures: positively described items and negatively described items. Among them, items 2 and 3 represent items with positive descriptions, and reverse scoring is used for these items. The higher the score, the greater the perception of stress. The items with negative descriptions include items 1 and 4, and reverse scoring is not used for these items. The total score of the four items represents the total score of perceived pressure. The higher the score, the greater the perceived pressure. The PSS-4 was confirmed to have good reliability in the Chinese population with a Cronbach's a coefficient of 0.833 [21].

#### Simplified coping style questionnaire (SCSQ)

The questionnaire is a self-assessment scale compiled by Xie Yaning and Zhang Yukun based on a foreign coping style scale and adapted according to the actual needs and the characteristics of the Chinese population [22]. The SCSQ consists of 2 subscales of positive coping and negative coping, including 20 items. The questionnaire is assessed using a 4-point scoring method as follows: not coping = 0, occasionally coping = 1, sometimes coping = 2, and often coping = 3. The positive coping subscale includes questions 1–12, which mainly reflect the characteristics of individuals adopting positive coping styles when encountering stress. The overall Cronbach's α of the scale was 0.90. The Cronbach's α coefficient of the positive coping subscale was 0.89, and the Cronbach's α coefficient of the negative coping subscale was 0.78.

#### Illness uncertainty scale (parent perceptions of uncertainty scale, PPUS)

The PPUS was revised by the domestic scholar Mai Jiaxuan [23] to assess the level of illness uncertainty of parents of hospitalized children. The scale contains 28 items and 4 dimensions, namely, ambiguity, lack of clarity, lack of information, and unpredictability. The scale uses a 5-point Likert scoring method, where strongly agree = 5 points, agree = 4 points, uncertain = 3 points, disagree = 2 points, and strongly disagree = 1 point. Items 6, 9, 11, 19, 23, and 25–28 are reverse scored, and a higher total score indicates a higher level of disease uncertainty. The scale has good construct validity. The Cronbach's α coefficient of the total scale is 0.91, and the Cronbach's α coefficients of each dimension range between 0.72 and 0.87.

#### Data collection methods

The study obtained the consent of the department leaders before analyzing the existing patient data in the department. The members of the research group selected the parents of the patients who met the inclusion and exclusion criteria to conduct the survey. When distributing the questionnaires, a unified guide was adopted to explain the content, purpose, significance, and confidentiality of the data to the subjects. For those who could not complete the questionnaires by themselves for various reasons, the investigator explained the above points to them one by one so that parents could decide whether to participate after understanding, and the investigator was responsible for completing the form. The questionnaires were collected on the spot. When the questionnaires were collected, the investigators checked the contents of the investigation. If there were any doubts, the parents were consulted, and the issue was resolved. If any problems, such as missing answers, were identified, the problems were corrected in a timely manner. A total of 108 questionnaires were distributed this time, 105 of which were valid. The effective recovery rate was 97.22%.

#### Statistical methods

The data were entered by two individuals into Excel, and the data were analyzed using SPSS 18.0 software. Enumeration data are described as the frequency and percentage; measurement data are expressed as the mean ± standard deviation. Influencing factors were assessed using t-test, analysis of variance, Spearman correlation analysis and multiple linear regression analysis, and *P* < 0.05 was considered statistically significant.

## Results

### General information

This study investigated 105 parents of children with SWS, of whom 90 were mothers (85.7%). Most of the participants were 35 years or older (37.1%), 68 lived in cities (64.8%), 98 were married (93.3%), and 74 (70.5%) had a high school education or above. Most (64.8%) family incomes could cover the medical expenses for children; 30 (28.6%) of respondents were anxious. In total, 22 (21.0%) of respondents screened positive for depression.

Among the 105 children with SWS who participated in the survey, the male to female ratio was close to 1:1. In total, 56 of respondents were only children (53.3%), and 66 were the first child (62.9%) in the family. Regarding affected eyes, 46 children each were affected in the left eye or right eye (43.8% each), and 13 children were affected in both eyes (12.4%). The results of univariate analysis revealed a statistically significant difference in the anxiety experienced by the children's parents regarding the uncertainty of the disease (*P* < 0.05). See Table 1 for details.

**Table 1:** Univariate analysis of illness uncertainty in parents of children with SWS (*n* = 105)

	**Characteristics**	**N (%)**	**Illness uncertainty score (x ± s)**	**F value/t value**	***P* value**
**Parents**	**Relationship to patient**				
	Father	15 (14.3)	77.60 ± 16.68	*T* = 0.462	0.645
	Mother	90 (85.7)	79.31 ± 12.68		
	**Age, years**				
	≤ 29	28 (26.7)	80.04 ± 13.16	*F* = 0.271	0.763
	29–35	38 (36.2)	77.82 ± 11.53		
	≥ 35	39 (37.1)	79.59 ± 14.99		
	**Residence**				
	City	68 (64.8)	77.81 ± 14.43	*T* = -1.45	0.150
	Town	37 (35.2)	81.38 ± 10.50		
	**Marital status**				
	Married	98 (93.3)	79.45 ± 13.44	*T* = 1.108	0.270
	Divorced	7 (6.7)	73.71 ± 9.16		
	**Education**				
	Junior high school and below	31 (29.5)	80.90 ± 10.33	*F* = 2.204	0.092
	High school or secondary school	22 (21.0)	82.23 ± 13.41		
	College or Undergraduate	48 (45.7)	75.75 ± 13.40		
	Master’s degree and above	4 (3.8)	87.25 ± 23.77		
	**Can family income cover children’s medical expenses?**				
	Yes	68 (64.8)	77.57 ± 12.94	*T* = -1.578	0.118
	No	37 (35.2)	81.81 ± 13.52		
	**Sleep quality**				
	Good	39	75.92 ± 13.66	*F* = 2.570	0.058
	General	62	80.65 ± 12.06		
	Insomnia	2	73.00 ± 16.97		
	Sleep disturbance	2	97.50 ± 26.16		
	**Anxiety**				
	GAD ≥ 10	30 (28.6)	86.53 ± 11.21	*T* = 3.896	0.000
	GAD < 10	75 (71.4)	76.08 ± 12.86		
	**Depression**				
	PHQ ≥ 3	22 (21.0)	83.36 ± 11.96	*T* = 1.728	0.087
	PHQ < 3	83 (79.0)	77.93 ± 13.40		
**Patient**	**Sex**				
	Male	58 (55.2)	78.57 ± 14.68	*T* = -0.426	0.671
	Female	47 (44.8)	79.68 ± 11.34		
	**Only child**				
	Yes	56 (53.3)	78.34 ± 12.44	*T* = -0.600	0.550
	No	49 (46.7)	79.90 ± 14.19		
	**Child's birth order**				
	First child	66 (62.9)	77.79 ± 12.36	*F* = 0.983	0.378
	Second child	33 (31.4)	80.73 ± 14.83		
	Third child	6 (5.7)	84.00 ± 13.52		
	**Affected eye**				
	Left eye	46 (43.8)	79.09 ± 14.14	*F* = 0.626	0.537
	Right eye	46 (43.8)	78.02 ± 12.30		
	Both eyes	13 (12.4)	82.69 ± 13.56		

### Illness uncertainty scores of parents of children with SWS

The total score of the child's parents' sense of disease uncertainty was 79.07 ± 13.24 points. The average item score was 2.82 ± 0.47 points. The scores of each dimension are ranked as follows from high to low: uncertainty, 33.55 ± 8.56 points; lack of clarity, 20.30 ± 4.37 points; unpredictable, 13.34 ± 3.26 points; and lack of information, 11.88 ± 2.76 points. See Table 2 for details.

**Table 2:** Total score and dimension scores of the uncertainty of parents of children with SWS (*n* = 105)

Characteristics	Number of entries	Minimum	Maximum	Score (x ± s)	Items are evenly divided (x ± s)	Score sort
Ambiguity	11	13	55	33.55 ± 8.56	3.05 ± 0.78	1
Lack of clarity	8	10	33	20.30 ± 4.37	2.54 ± 0.55	2
Not predictable	4	4	20	13.34 ± 3.26	3.34 ± 0.82	3
Lack of information	5	6	21	11.88 ± 2.76	2.38 ± 0.55	4
Total score	28	42	116	79.07 ± 13.24	2.82 ± 0.47	_

### Correlations between illness uncertainty, stress perception and simple coping among parents of children with SWS

Illness uncertainty was positively correlated with stress perception (*r* = 0.304, *P* < 0.01) and negatively correlated with simple coping (*r* = -0.220, *P* < 0.05). The correlation of each dimension is shown in Table 3.

**Table 3:** Correlation analysis (*r* value) between illness uncertainty and perception of stress and simple coping in parents of children with SWS

**Characteristics**	**Perception of stress**			**Simple coping**		
	**Positive**	**Negative**	**Total score of stress perception**	**Positive**	**Negative**	**Total score of simple coping**
**Ambiguity**	-0.004	0.410**	0.313**	-0.259**	0.076	-0.185
**Lack of clarity**	0.087	0.134	0.131	-0.253**	-0.052	-0.227*
**Not predictable**	0.143	0.126	0.178	-0.187	-0.062	-0.180
**Lack of information**	0.137	-0.015	0.062	0.115	-0.013	0.083
**Disease indeterminate total score**	0.074	0.347**	0.304**	-0.266**	0.011	-0.220*

^*^P < 0.05

^**^P < 0.01

### Influencing factors of illness uncertainty in parents of children with SWS

Multiple linear regression analysis was performed using illness uncertainty of parents of children with SWS as the dependent variable and item anxiety, stress perception and simple coping, which exhibited statistically significant univariate analysis results, as independent variables. The results showed that the influencing factors of illness uncertainty of the parents of the children included anxiety and simple coping. The independent variable assignments are shown in Table 4, and the results of multiple regression analysis are shown in Table 5.

**Table 4:** Assignment methods of independent variables that affect the factors affecting illness uncertainty in parents of children with SWS

Characteristics	Assignment method
Anxiety	Positive = 1; Negative = 0
Perception of stress	Substitute the original value
Simple coping	Substitute the original value

**Table 5:** Results of multiple regression analysis of factors affecting illness uncertainty in parents of children with SWS

**Characteristics**	B	SE	β	T value	*P* value
Constant term	83.449	6.845	-	12.191	0.000
Anxiety	10.855	2.985	0.372	3.637	0.000
Perception of stress	0.117	0.650	0.019	0.180	0.858
Simple coping	-0.266	0.120	-0.212	-2.214	0.029

*R* = 0.419; adjusted R-squared = 0.176; F = 7.172; *P* = 0.000

## Discussion

### Parents of children with SWS exhibit a high level of illness uncertainty

The results of this survey showed that the total score of the parents’ sense of illness uncertainty was achieved in greater than 50% of the total score of items, indicating that the sense of lack of disease certainty was at a high level [24]. Previous studies have found that parents of children with brain tumors [25], idiopathic membranous nephropathy [14], and Kawasaki disease [26] also have a high sense of disease uncertainty. These findings may be related to the unpredictable treatment effect and prognosis as perceived by children's parents as well as parents' limited understanding of the disease. The mode of modern medical care has changed from patient-centered therapy to patient and family-centered therapy. The recipients of care are not only patients but also their families. Parents with a high degree of disease uncertainty do not have an appropriate level of coping after the onset of the disease, which is not conducive to the patient's health. Therefore, it is of great significance to analyze the relevant factors that cause disease uncertainty in patients’ parents. As noted in the table, the item with the highest average score is the dimension of “uncertainty”. The reason for this finding is that the parents of the children were relatively lacking in disease-related knowledge. Therefore, during hospitalization, medical staff should adopt a variety of information support methods [27], frequently communicate with the parents of patients through multiple channels, actively evaluate parents' disease uncertainty, and strengthen health guidance to reduce parents' anxiety and illness uncertainty.

### The main influencing factors of illness uncertainty in parents of children with SWS

#### Anxious parents of children with SWS have a more obvious sense of illness uncertainty

The results of the study show that the anxiety of parents of children with SWS is an influencing factor of parents' sense of disease uncertainty. The more anxious the parents of the children are, the stronger their sense of disease uncertainty. Zhu Tingting et al. [28] studied the parents of 113 children with primary nephrotic syndrome and found that the anxiety score of parents was higher than the domestic norm and was positively correlated with parents’ illness uncertainty. These findings are similar to the results of this study. In this study, 62.9% of the children were first-born children in their families. In addition to the complexity of the disease and the prolonged treatment cycle, this may lead to parents' anxiety and negative emotions. When they are anxious, parents have no time to pay attention to the relevant information of the disease. This anxiety directly leads to the occurrence of a sense of disease uncertainty. For the patient's parents, the material, spiritual and information support provided by relatives, friends, and medical staff can relieve anxiety and directly reduce disease uncertainty [29]. This finding suggests that medical staff should communicate with patients’ families in a timely manner; pay attention to patients and their families [30]; strengthen health education, communication and emotional counseling for parents; guide parents to actively seek social support; improve parents’ psychological resilience, and actively help parents. These interventions can effectively reduce the level of disease uncertainty through the use of internal resources of the family to adjust the parents’ psychological state and enhance the resilience of the family [11].

#### A positive coping style can reduce illness uncertainty in children’s parents

This study shows that the coping style of the patient's parents is an influencing factor of parents' sense of disease uncertainty. The more active the parents of the patient are, the lower the sense of disease uncertainty. de Hosson M et al. [12] also obtained similar results regarding parents' uncertainty and coping styles in their study of children with congenital heart disease after surgery. Some researchers also found that the higher the educational level of family members, the lower the disease uncertainty [31, 32]. Among the respondents of this survey, parents’ educational level was relatively high. These parents employed a more positive coping approach. Positive coping methods are more conducive to producing positive results than negative coping methods. Parents acquire disease-related knowledge from various sources through active medical treatment and can view the development process, prognosis and outcome of the patient's disease more rationally and objectively, thereby preventing disease uncertainty. These parents report low uncertainty. This finding suggests that medical staff should strengthen the assessment of the coping style of parents of children with SWS. For those who are accustomed to adopting negative coping styles, information support in terms of disease diagnosis and treatment, prognosis, and care should be improved [13], and effective preoperative guidance and treatment should be provided. Guide [33] promoted the formation of an effective positive coping style, and those who tend to use positive coping styles should focus on the exercise of their personal abilities, tap their own potential and strength, and reduce disease uncertainty. Huang Zhirong et al. [34] found in a study of parents of premature infants that family empowerment programs can reduce illness uncertainty in parents of premature infants and improve family care readiness and postdischarge coping abilities. In the future, we will perform research focused on interventions for parents of children with SWS.

### Limitations

This study also had some limitations that are likely to affect the generalizability of the results. The sample size of the study was relatively small, and the sample size of parents of children with SWS should be expanded in future studies. The research object of this study was parents of children with SWS, and the sample size may be expanded in the future to conduct research on the child's family members to make the research results more representative.

## Conclusions

In Sturge‒Weber syndrome, the greatest uncertainty is still derived from the treatment of ocular complications. The treatment provided for glaucoma often needs to last for life. At the same time, seizures are the most common neurological manifestation and typically present in the first months of life. In addition, vascular malformations in the eyeball may also develop with age. In view of this information, our study focused with patients with Sturge‒Weber syndrome who had received eye treatment and obtained relatively clear research results. For patients with high disease uncertainty, we have also taken corresponding measures in clinical diagnosis and treatment. For example, the pioneering inclusion of these patients into the chronic eye disease management system allows physicians to pay close attention to the development of the disease but also enables patients to get help from doctors or social workers anytime and anywhere, which may reduce their disease uncertainty level and improve their quality of life. Parents of children with SWS have a high level of illness uncertainty, and this uncertainty is related to parents’ anxiety level and coping style. Therefore, medical staff should correctly assess the level and influencing factors of parents’ illness uncertainty and provide targeted intervention measures to reduce disease uncertainty.

The results of this study are based on data obtained from a research center in China. When the results of this study are extended to non-Chinese populations, cultural differences must be taken into account.

## Data Availability

All data analyzed during this study are included in this article. Further enquiries can be directed to the corresponding author.
