# Correction: Current situation and influencing factors of Disease uncertainty in parents of children with Sturge‒Weber syndrome: a retrospective study

**DOI:** 10.1186/s12887-023-04374-7

**Published:** 2023-10-28

**Authors:** Na Du, Yue Wu, Shanshan Xiong, Hong Ji, Lulu Huang, Wenyi Guo, Changjuan Zeng

**Affiliations:** 1 Department of Nursing, Ninth People?s Hospital, Shanghai Jiao Tong University School of Medicine, Shanghai, 200011 China; 2 Department of Ophthalmology, Ninth People?s Hospital, Shanghai Jiao Tong University School of Medicine, Shanghai, 200011 China; 3 Shanghai Key Laboratory of Orbital Diseases and Ocular Oncology, Shanghai, 200011 China; 4 Shanghai JiaoTong University School of Nursing, Shanghai, 200025 China

**Correction: BMC Pediatr 23, 64 (2023)**

10.1186/s12887-023-03857-x

Following publication of the original article [1], the authors identified that the Award Number of the Funding in the Funding section was miswritten in the pubished version as “No.JYLJ201905” in the statement “Clinical Research Program of 9th People’s Hospital afliated to Shanghai Jiao Tong University School of Medicine(No.JYLJ201905)” which should be corrected as “(No.JYLJ201904)”.
